# *Brevundimonas vesicularis* sepsis in a 2-month-old infant in rural Gambia: a case report

**DOI:** 10.1186/s13256-025-05567-7

**Published:** 2025-10-21

**Authors:** Minteh Molfa, Williams Oluwatosin Adefila, Baleng Mahama Wutor, Momodu Keita, Yusuf Abdulsalam Olawale, Mayowa Banke Omotosho, Ousman Barjo, Rasheed Salaudeen, Isaac Osei, Muhammed Wally, Grant Mackenzie

**Affiliations:** 1 Medical Research Council Unit The Gambia at London School of Hygiene and Tropical Medicine, P.O. Box 273, Banjul, The Gambia; 2 Faculty of Infectious and Tropical Diseases, London School of Hygiene and Tropical Medicine, London, UK; 3 Murdoch Children’s Research Institute, Melbourne, Australia; 4 Department of Paediatrics, University of Melbourne, Parkville, Australia

**Keywords:** Case report, *Brevundimonas vesicularis*, Sepsis, Congenital heart disease, Opportunistic infections

## Abstract

**Background:**

*Brevundimonas vesicularis,* a Gram-negative bacillus and non-lactose fermenter, is primarily found in both clinical and environmental samples. Although it rarely causes infections and is typically regarded as an opportunistic pathogen, it has been associated with cases of bacteremia, peritonitis, meningitis, arthritis, and skin infections. While there is high variability among cases, depending on site, infection severity, patient age, immune status, and geographic location, most cases occur in patients with underlying congenital conditions and immunocompromised individuals, which could represent an emerging global concern, particularly in low- and middle-income countries.

**Case presentation:**

We present a 2-month-old African male infant who exhibited signs of sepsis and cyanotic congenital heart disease. The blood culture identified *Brevundimonas vesicularis*. Antimicrobial susceptibility testing was performed, and the bacterium was found to be sensitive to all the antibiotics evaluated. We treated the child with the empiric first-line antibiotics intravenous amoxicillin clavulanate and intravenous gentamicin for 6 days. The child improved and was referred for an echocardiogram and cardiology review.

**Conclusion:**

This report details a rare case of *B. vesicularis* sepsis in a 2-month-old infant with congenital heart disease in the Gambia. *B. vesicularis* is an emerging pathogen linked to opportunistic infections in patients with congenital disorders and immunocompromised individuals. This report highlights comorbidities as key risk factors for acquiring *B. vesicularis* infection, underscoring the need for a multidisciplinary and holistic approach to improve clinical outcomes. Therefore, an improved surveillance system, skilled personnel, and adequate infrastructure are essential for early detection, accurate diagnosis, and effective management based on local antibiogram data, to control antibiotic resistance.

## Introduction

*Brevundimonas vesicularis* belongs to the Alphaproteobacteria class and Caulobacteraceae family [1], which was previously assigned to *Pseudomonas vesicularis* but later reclassified into a new genus “*Brevundimonas*” by Segers *et al*. in 1994 [2]. It is an aerobic, non-lactose, oxidase- and catalase-positive, flagellated Gram-negative rod that can be isolated from both environmental and clinical specimens [3]. The organism can be isolated in the laboratory on blood or chocolate agar. However, not all strains grow on MacConkey agar [4].

It is an opportunistic pathogen that is associated with septicemia, pneumonia, meningitis, septic arthritis, urinary tract infection in children, and cervicitis in women [5–7]. It mainly infects patients with underlying health conditions such as congenital heart disease, respiratory distress syndrome, multiple congenital cerebral anomalies, and meconium aspiration syndrome [8]. It affects patients with foreign bodies and immunocompromised individuals but rarely those without predisposing disease [5, 9]. Many of these instances of infection were either hospital- or community-acquired [1, 6].

*B. vesicularis* is an emerging global opportunistic pathogen of concern. However, a few invasive *B. vesicularis* infections exist in literature, and no case in the Gambia has yet been reported [1, 6–12]. A case series of *B. vesicularis* infections in the neonatal period at a tertiary health facility in Turkey reported that, of the eight cases, four patients had congenital heart disease, two had respiratory distress syndrome, multiple congenital cerebral anomalies (1), and meconium aspiration syndrome (1). All eight cases had septicemia [8]. In rural Gambia and other resource-limited settings, healthcare infrastructure is often inadequate, restricting access to diagnostic tools and effective treatments. *B. vesicularis* infections can worsen existing health issues, such as malnutrition and coinfections with other pathogens, resulting in higher morbidity and mortality rates in such settings [1]. The emergence of opportunistic infections such as *B. vesicularis* in vulnerable groups highlights the need for better surveillance and healthcare approaches. Such infections highlight broader global health challenges, including access to healthcare. Recognizing and addressing the impact of *B. vesicularis* infections in resource-constrained environments is vital for improving public health and reducing health disparities globally [13].

Here, we report a case of *B. vesicularis* sepsis in a 2-month-old child with cyanotic congenital heart disease at a hospital in rural Gambia.

## Case presentation

A 2-month-old African male infant was brought by the mother on account of a 2-week history of nonparoxysmal cough, fast breathing, and lower-chest-wall indrawing. There was fever, and the mother complained that the child was highly irritable at first but later became poorly responsive. The respiratory distress worsened with feeding, and the mother noticed the child’s lips had turned blue. There was no history of choking on feeds, vomiting, or diarrhea. There was no history of forced feeding or giving the child herbal concoctions. The mother also noticed that the child had not gained appreciable body weight since birth. The pregnancy was planned and progressed without complications, with the mother consistently attending antenatal care (ANC) appointments. It was a single-fetus pregnancy, and the baby was born at full term. The mother had no notable previous medical conditions. She received the appropriate prenatal tetanus toxoid vaccination, and her human immunodeficiency virus (HIV) screening result was negative. No maternal risk factors were present, and she delivered at the hospital through spontaneous vaginal delivery. The newborn had a birth weight of 3 kg, and the immediate postpartum condition was stable. Both the mother and baby were discharged on the day of delivery. The infant was exclusively breastfed.

A week before presenting to our facility, the child developed respiratory symptoms and was taken to a primary healthcare center, where he was treated as an outpatient and received oral medications, including amoxicillin, paracetamol, and vitamin C syrup. However, the child’s condition did not improve, and he was then taken to our facility by his mother.

Physical examination revealed an acutely ill-looking, lethargic infant with a temperature of 35.10 °C, in respiratory distress and with central cyanosis. He was, however, anicteric, not pale, and was well hydrated. The capillary refill time was 2 seconds.

The respiratory rate was 65 breaths per minute. Oxygen saturation was < 85%, and there was marked chest retraction and bronchial breath sounds on auscultation. The chest was resonant on percussion.

The heart rate was 140 beats per minute, with a continuous murmur in the sternal region. Other systemic examinations were unremarkable.

A diagnosis of sepsis with cyanotic congenital heart disease with a differential diagnosis of pneumonia was made. The investigations requested included a blood culture and antibiotic susceptibility testing, a rapid diagnostic test (RDT) for malaria, and hemoglobin concentrations. We had wanted to perform a complete blood count, electrolyte assay, chest X-ray, and echocardiogram, but these services were not available at our facility. The hemoglobin was 13.2 g/dl, the RDT for malaria was negative, and the random blood sugar was 4.1 mmol/l. After collecting the blood culture samples, we commenced the child on the first-line empirical antibiotics per national guidelines: intravenous amoxicillin clavulanic acid 45 mg/kg 12-hourly and intravenous gentamicin 5 mg/kg daily. To prevent hypoglycemia, a 10% dextrose solution was given. We placed the child on humidified oxygen via nasal prong 1 l/minute and kept the child warm. Feeding was via a nasogastric tube using expressed breast milk.

## Laboratory analysis

The blood sample collected was processed and cultured on blood agar, chocolate agar, and MacConkey agar plates. After 24 hours at standard temperature, yellow-colored colony growth was observed on both blood and chocolate agar. After 48 hours of incubation, Gram-negative short oxidase-positive rods were isolated. Using the analytical profile index (API) 20NE, with code no. 1460204, we identified *Brevundimonas vesiculari.* The antibiotic sensitivity was assessed using the Kirby–Bauer disk diffusion method on Mueller–Hinton agar. Using the Clinical and Laboratory Standards Institute’s guidelines, the bacterium was sensitive to all the antibiotics tested including ampicillin, amoxicillin–clavulanic acid, ceftriaxone, ceftazidime, cefotaxime, cefoxitin, gentamicin, ciprofloxacin, chloramphenicol, and cotrimoxazole [14].

## Progress report

After 48 hours of hospital admission, the body temperature was between 36.70 and 38.50 °C. The child was well hydrated, and the oxygen saturation had improved to 92%. The child was still in respiratory distress. On the basis of the culture and sensitivity results as well as the improvement in the child’s condition, we continued the intravenous antibiotics unchanged. After 72 hours of management, the body temperature was between 36.50 and 37.20 °C. The child was well hydrated, conscious, and alert. The oxygen saturation was between 88% and 92% while on oxygen. A total of 6 days after hospital admission, the fever had subsided. The central nervous system examination revealed increased consciousness, and the child was alert and was now feeding (expressed breast milk [EBM]) using a cup and spoon. A repeat blood culture showed no bacterial growth. We then referred the child to the teaching hospital for an echocardiogram and cardiologist review.

## Discussion

*Brevundimonas vesicularis* is a non-lactose fermenter, Gram-negative bacillus, and is usually isolated from clinical and environmental samples. It is an opportunistic pathogen that affects children and adults. Most cases may be due to underlying diseases or predisposing factors. There is high variability among the cases depending on the site and severity of infection, the age and immune status of the patients, the background health condition, and the geographic location. However, a significant proportion of *B. vesicularis* infections are found in patients with comorbidities and those who are immunocompromised [8–10, 15].

It is difficult to determine the exact global incidence of *B. vesicularis* causing sepsis owing to several factors, including underdiagnosis and limited data. However, about 49 cases isolated between 2001 and 2010 have been documented in the literature [1].

There are limited reports of *B. vesicularis* infections in the literature [12]. The limited number of reported cases of *B. vesicularis* infection leaves a void in understanding the spectrum of diseases caused by this pathogen and the optimal treatment regimens [9]. To the best of our knowledge, few cases of *B. vesicularis* sepsis have been reported in the literature from Africa, and no case in the Gambia has yet been reported.

*Brevundimonas* species have emerged as opportunistic pathogens, with documented cases of infection in the blood and at various anatomical sites, including the skin and soft tissues, the urinary tract, liver, meninges, and peritoneum [16]. The precise factors predisposing individuals to *B. vesicularis* infection remain unclear. However, a significant association exists with immunocompromised states. *B. vesicularis* infection is common in children with an underlying disease such as congenital heart disease, chronic kidney disease, diabetes mellitus, sickle cell anemia, leukemia, myelodysplastic syndromes, systemic lupus erythematosus, glomerulopathy, and cystic fibrosis, owing to immunological defect [17].

Our patient had a suspected congenital heart disease, which could have predisposed him to *B. vesicularis* infection. The abnormal blood flow associated with congenital heart diseases as well as pulmonary ventilation–perfusion mismatch increases the risk of opportunistic infections [18]. In addition, the chronic inflammation associated with congenital heart disease can alter immune responses, making it easier for infections to occur [19].

Bacterial growth was identified after 48 hours of incubation of the blood sample in the BacT/ALERT 3D machine. However, no growth was seen on the MacConkey agar plate until another 48 hours of subsequent incubation. Because *B. vesicularis* grows slowly on media such as MacConkey agar, it is often necessary to incubate it for a longer period [6, 7]. Such prolonged periods of incubation often delay antibiotic susceptibility testing and feedback for clinical decision-making. The child was treated with the first-line empirical antibiotics for 6 days per national guidelines, and a repeat blood culture was negative. In our case, *B. vesicularis* was sensitive to all the antibiotics that were tested, namely, ampicillin, amoxicillin–clavulanic acid, ceftriaxone, ceftazidime, cefotaxime, cefoxitin, gentamicin, ciprofloxacin, chloramphenicol, and cotrimoxazole [Table 1]. However, *B. vesicularis* antibiotic susceptibility pattern varies widely depending on the geographical location, invasiveness, and virulence [15].

**Table 1:** Results of laboratory investigations

Laboratory investigation	Result	
Malaria RDT	Negative	
Hemoglobin	13.2 g/dl	
API 20NE code number	1,460,204 (*Brevundimonas vesiculari* 99.9)	
Sensitivity	Antibiotic	Sensitivity result
	Ceftriaxone	Sensitive
	Ciprofloxacin	Sensitive
	Cefoxitin	Sensitive
	Gentamicin	Sensitive
	Ceftazidime	Sensitive
	Cefotaxime	Sensitive
	Cotrimoxazole	Sensitive
	Amoxicillin-clavulanic acid	Sensitive
	Ampicillin	Sensitive
	Chloramphenicol	Sensitive
	Tetracycline	Sensitive

Most *B. vesicularis* infections are sensitive to macrolides, penicillin, cephalosporin, tetracycline, and chloramphenicol [3–8, 11]. The carbapenem group of antibiotics, including meropenem, imipenem, and doripenem, has been reported to be effective in the treatment of *B. vesicularis* infections. Resistance of *B. vesicularis* to ciprofloxacin ceftazidime, cefoxitin, cotrimoxazole, tobramycin, netilmicin, amoxicillin–clavulanic, piperacillin and ticarcillin, and cefepime has been reported in various studies (Table 2) [6–12, 20–23]. The absence of antibiotic resistance in our case may be due to the lack of genetic determinants for resistance or environmental factors. Consequently, establishing an empiric antibiotic regimen against *B. vesicularis* infections remains a challenge owing to limited data availability [10, 12, 23].

**Table 2:** Antimicrobial resistance patterns of *Brevundimonas vesicularis* cases from 1977 to 2015

Authors, year	Country	Resistant to	Sensitive to
S.M. Bhatawadekar, *et al*., 2011	India	Ceftazidime, cefoxitin, cotrimoxazole, tobramycin, netilmicin	Amoxicillin, piperacillin, ticarcillin, first-generation cephalosporins, imipenem meropenem, amikacin, gentamicin, ciprofloxacin, cefotaxime
A. Ben Haj Khalifa *et al*., 2012	Tunisia	Piperacillin and ticarcillin	Cefotaxime, ceftazidime, imipenem, aztreonam, piperacillin/tazobactam, gentamicin, amikacin, ofloxacin, la ciprofloxacin
Shih-Ta Shang *et al*., 2011	Taiwan	Ampicillin, ciprofloxacin, ceftazidime, cefepime	Aminopenicillins, antipseudomonal penicillins, cephalosporins, carbapenems, aminoglycosides,
A.M. Planes *et al*., 1992	Spain	Piperacillin and ceftazidime	Ampicillin, cefazolin, cefoxitin, cefotaxime, ceftriaxone, gentamicin, tobramycin, amikacin, doxycycline, fosfomycin, chloramphenicol, co-trimoxazol
L. Anne Otto *et al*., 1977	USA	Colistin, nalidixic acid, sulfisoxazole	Gentamicin, kanamycin, cephalothin, tetracycline, ampicillin, streptomycin
N. Karadag *et al*. (eight cases), 2012	Turkey	All the cases were resistant to piperacillin–tazobactam, ceftazidime, and aztreonam	All the cases were sensitive to aminoglycosides and third-generation cephalosporin

## Conclusion

It is significant to note that *B. vesicularis* may be more common than reported in literature, and it is crucial to conduct further research on this emerging low-virulent opportunistic pathogen. Moreover, it is vital to investigate and report findings on *B. vesicularis* infections in both immunocompromised and immunocompetent patients to further increase our knowledge about the pathogen’s virulence and distribution. This report shows that comorbidities are essential risk factors in acquiring *B. vesicularis* infection. Hence, a multidisciplinary and holistic approach may be required in the management of patients with *B. vesicularis* infection. This will further improve and boost clinical outcomes. Finally, early detection and management based on local antibiogram data are essential to limit antibiotic resistance. The lack of microbiology services in many settings in sub-Saharan Africa will mean that infections with *B. vesicularis* are underdiagnosed and may be poorly treated.

## Data Availability

Data sharing is not applicable to this article as no datasets were generated or analyzed during the current study.

## Abbreviations

RDT: Rapid diagnostic test
IV: Intravenous
API: Analytical profile index
NE: Non-Enterobacteriacea
CNS: Central nervous system
EBM: Evidence-based medicine
B: *Brevundimonas*
